# Respiratory Mucosal Proteome Quantification in Human Influenza Infections

**DOI:** 10.1371/journal.pone.0153674

**Published:** 2016-04-18

**Authors:** Tony Marion, Husni Elbahesh, Paul G. Thomas, John P. DeVincenzo, Richard Webby, Klaus Schughart

**Affiliations:** 1 University of Tennessee Health Science Center, Department of Microbiology, Immunology and Biochemistry, Memphis, United States of America; 2 Department of Immunology, St. Jude Children’s Research Hospital, Memphis, United States of America; 3 University of Tennessee Health Science Center, Department of Pediatrics, Memphis, United States of America; 4 Children’s Foundation Research Center at Le Bonheur Children’s Hospital, Memphis, United States of America; 5 Department of Infectious Diseases, St Jude Children’s Research Hospital, Memphis, United States of America; 6 Department of Infection Genetics, Helmholtz Centre for Infection Research, Braunschweig, Germany; 7 University of Veterinary Medicine Hannover, Hannover, Germany; Indiana University, UNITED STATES

## Abstract

Respiratory influenza virus infections represent a serious threat to human health. Underlying medical conditions and genetic make-up predispose some influenza patients to more severe forms of disease. To date, only a few studies have been performed in patients to correlate a selected group of cytokines and chemokines with influenza infection. Therefore, we evaluated the potential of a novel multiplex micro-proteomics technology, SOMAscan, to quantify proteins in the respiratory mucosa of influenza A and B infected individuals. The analysis included but was not limited to quantification of cytokines and chemokines detected in previous studies. SOMAscan quantified more than 1,000 secreted proteins in small nasal wash volumes from infected and healthy individuals. Our results illustrate the utility of micro-proteomic technology for analysis of proteins in small volumes of respiratory mucosal samples. Furthermore, when we compared nasal wash samples from influenza-infected patients with viral load ≥ 2^8^ and increased IL-6 and CXCL10 to healthy controls, we identified 162 differentially-expressed proteins between the two groups. This number greatly exceeds the number of DEPs identified in previous studies in human influenza patients. Most of the identified proteins were associated with the host immune response to infection, and changes in protein levels of 151 of the DEPs were significantly correlated with viral load. Most important, SOMAscan identified differentially expressed proteins heretofore not associated with respiratory influenza infection in humans. Our study is the first report for the use of SOMAscan to screen nasal secretions. It establishes a precedent for micro-proteomic quantification of proteins that reflect ongoing response to respiratory infection.

## Introduction

Each year, about 500 million people are infected by the influenza A virus (IAV) worldwide, of which about 500,000 die [1]. In recent history, the emergence of new influenza subtypes has caused several pandemics [2–4]. The most severe pandemic in 1918 caused about 30–50 million deaths worldwide [5], and a new variant of a seasonal H1N1 virus, pH1N1, caused a world-wide pandemic in 2009 [6–8]. Avian viruses can also directly infect humans. In particular, two subtypes, H5N1 and H7N9, may cause severe disease with lethal outcome [9–13]. Adverse health conditions, such as obesity and diabetes, and genetic make-up predispose influenza patients to more severe forms of the disease [14–19]. Cytokines and chemokines released in a cytokine storm in response to influenza infection contribute to disease severity [20]. Unraveling the pathogenesis of influenza in humans so as to identify potential targets for human therapeutics and predictors of disease severity necessitates the evaluation of the main site of viral replication, the mucosal tissues of the respiratory tract. The majority of the disease pathogenesis caused by influenza occurs after viral replication has already started to decline [21], thus adding to the impetus to develop host-response-targeted therapies in addition to continuing evaluation of better antiviral therapeutics. Additionally, host-response-based diagnostics may improve identification of patients at highest risk of disease progression. Quantitative mucosal biomarker identification is important for such work to proceed rationally.

Most multiplex assays for disease protein biomarkers in inflammation and infection have been limited to detection of chemokines and cytokines expected to play a role in disease pathogenesis and for which prepared kits are readily available. Hence those assays have inherent bias. Here, we have attempted a new approach to biomarker identification in influenza infected patients using an aptamer-dependent, micro-proteomic approach (SOMAscan^®^). SOMAscan is a recently developed technology that can simultaneously quantify more than 1,000 human proteins in small volumes of complex biological fluids [22]. We used the SOMAscan version 1.2k that had a multiplex library of 1,129 SOMAmers (Slow Off-rate Modified nucleic acid based Aptamers) that each quantifies a single soluble protein [23]. SOMAscan transforms the number of each protein-bound SOMAmer into a quantitative measure of protein concentration [24]. SOMAscan is highly sensitive with a threshold of detection of 30 femtomolar, <1 pg/ml and 10^8^-fold dynamic range for quantification of proteome changes in mice [25] and humans [22, 24, 26, 27].

Here, we identified several differentially expressed proteins in mucosal secretions that heretofore have not been associated with respiratory influenza infection in humans. Our results indicate that SOMAscan is well suited for biomarker discovery in respiratory infections.

## Results

### SOMAscan detects a broad spectrum of proteins in mucosal secretions

We first evaluated whether an unbiased proteomic screen like SOMAscan would identify proteins in mucosal samples other than those detected in standard cytokine and chemokine assays [28, 29] and already known to be relevant for inflammation and immune response to respiratory influenza infection. For this, we analyzed 24 nasal wash samples from a previously described influenza cohort [28] (S1 Table). The samples included 18 from patients with identified influenza infection, 11 patients infected with IAV H3 strain, two infected with IAV pH1 subtypes, and five infected with influenza B. The healthy control group consisted of samples from six individuals who were not infected with influenza, who showed no clinical signs of infection, such as fever, cough, or sore throat [28] and who appeared to be otherwise healthy. Viral loads in infected patients, determined by quantitative reverse transcription PCR (qRT-PCR) [28], ranged from 0.72 to 5.1x10^6^ copies/ml [28].

**Fig 1:**
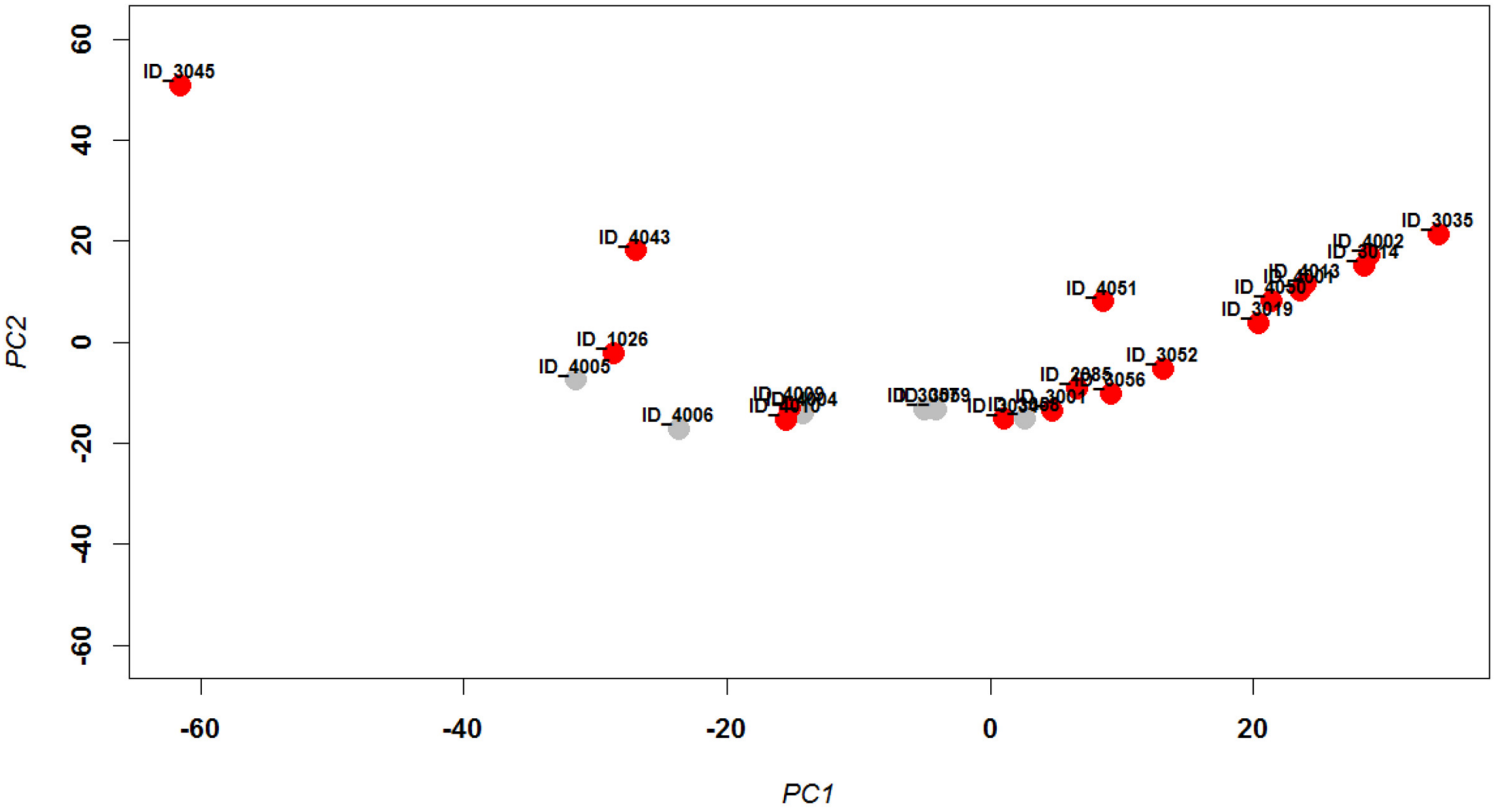
PCA analysis of normalized protein expression values. Principle component analysis (PCA) was performed with quantile normalized log_2_–transformed protein expression values from nasal washes for all 24 samples. The first two principal components are shown representing 24% and 17%, respectively, of the total variation. Healthy controls are labeled gray and IAV-positive samples (in which influenza A or B was detected by PCR) are labeled red. In addition, sample identities (e.g. ID_4043) are shown. Horizontal and vertical axis represent principle component 1 and 2, respectively.

The nasal washes were analyzed with the 1.2k version of SOMAscan with 1,129 SOMAmers that simultaneously quantified 1,030 different human proteins [24]. Quantitative expression signals were log_2_-transformed and quantile-normalized and then examined by principle component analysis (PCA) to visualize variation and grouping of samples. PCA is a mathematical transformation that reduces variation in a large data set to a few dimensions that project differences determined by the strongest variables. The first two dimensions that represent the highest variations of a PCA analysis can be visualized in a 2D plot. In this way, samples that are most disparate with respect to protein expression levels are found more distantly located from each other in the 2D PCA plot, and samples with similar protein expression levels are closer to each other in the plot. As shown in Fig 1, most infected patients (red circles) segregated together indicating that their protein expression levels were similar. Most influenza virus-positive patients segregated to the right of PCA1 = 0 suggesting that these patients had similar changes in their mucosal proteome compared to others in the cohort. The healthy controls (grey circles) segregated to the left except for ID_3058 that segregated with the leftmost group of virus-infected patients. Three patients who were diagnosed as virus-positive (red circles), based on qRT-PCR [28], segregated with healthy controls (grey circles). Patient ID_3045 segregated independently of all other patients. The imperfect PCA distribution of infected versus healthy patients is in part due to the small sample size but also reflects the large uncontrollable heterogeneity which is an intrinsic property of human cohorts. Here, many confounding factors, such as adverse health conditions, time after infection, genetics, and general environment, may influence mucosal protein expression levels and are difficult or impossible to control.

There was good correlation between measurements in SOMAscan for four analytes that were previously quantified by Luminex in samples from the same patients [28]. Relative protein expression levels for IL-6, CXCL10/IP10, CCL7/MCP3, and CXCL8/IL-8 were highest among proteins measured by SOMAscan, and those levels correlated well with measurements of the same proteins by Luminex (S1 Fig). The majority of proteins quantified by SOMAscan were not measured in the previous Luminex study. The correlation between Luminex and SOMAscan validates our results and verifies SOMAscan's potential to identify proteins relevant to pathophysiology, inflammation, and immune response in mucosal secretions after influenza infection.

### Differentially expressed proteins in influenza virus infected patients

**Fig 2:**
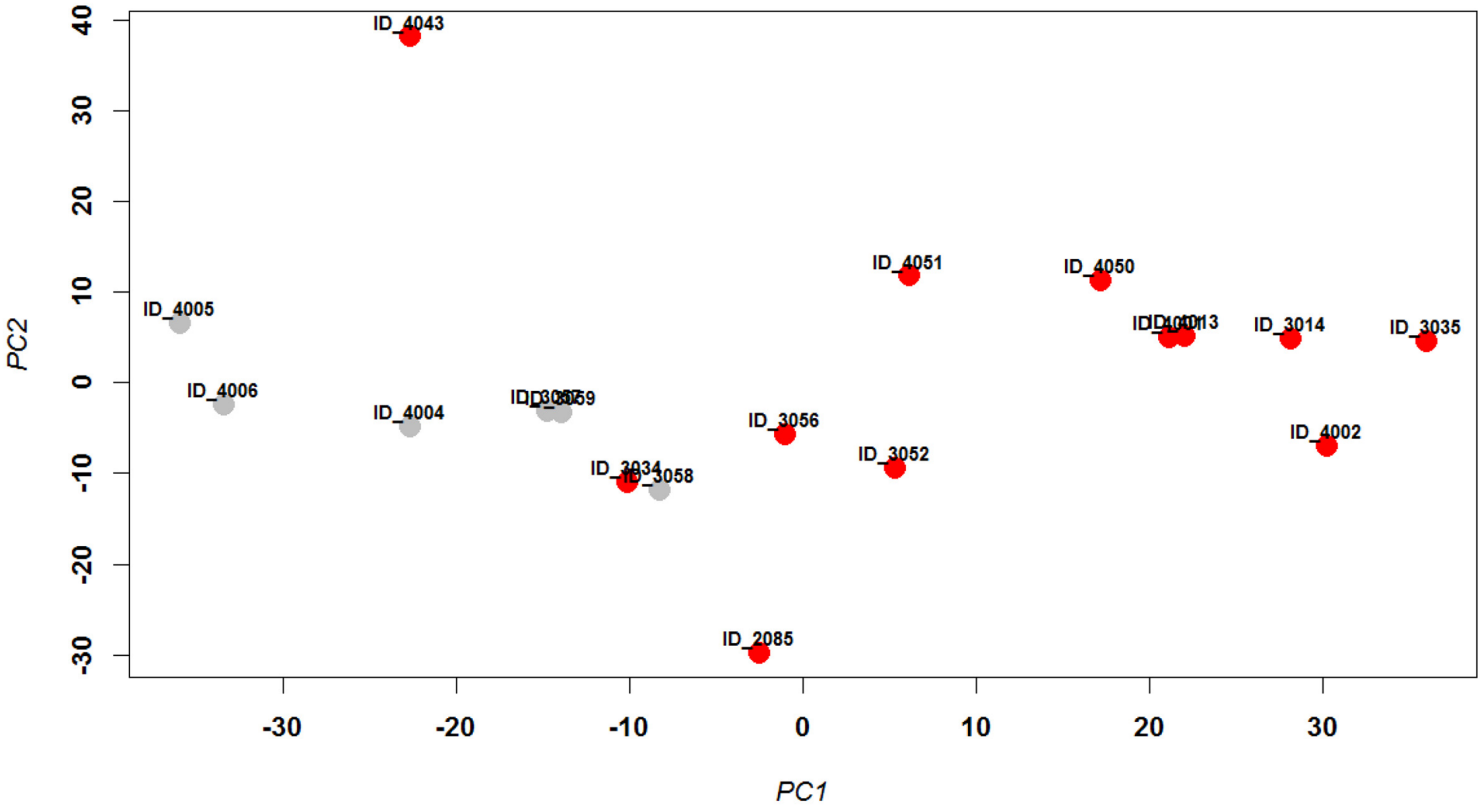
PCA analysis of normalized protein expression values of subset A. Principle component analysis (PCA) was performed with normalized log_2_–transformed protein expression values from nasal washes for 18 samples from the subset A that was selected based viral load being higher than > 2^8^. The first two principal components are shown representing 42% and 16%, respectively, of the total variation. Healthy controls are labelled gray and IAV-positive samples are labelled red. In addition, sample identities (e.g., ID_4002) are shown. Horizontal and vertical axis represent principle component 1 and 2, respectively.

A comparison of all 18 samples from infected patients with the six samples from healthy individuals did not identify statistically significant differentially expressed proteins (DEPs). This result is consistent with the incomplete segregation of positive and negative samples in the PCA (Fig 1) and previous chemokine and cytokine measurements in samples from the entire patient cohort from which the samples analyzed herein were obtained [28]. The low viral load in some of the infected patients (S1 Table) may account for this result. It is well known from animal experiments that immune responses and pathologies depend largely on viral loads (reviewed in [30]). Furthermore, the distribution of viral loads among all of the infected patients in the entire patient cohort [28] was bimodal (S2 Fig). Based upon the bimodal distribution between patients with higher versus lower viral RNA levels in the entire influenza infected patient cohort, we selected a subset of patients with a minimum viral load of 2^8^ (subset A, S2 Table) to identify proteins that were differentially expressed between infected patients (12 samples) and healthy controls (6 samples). Fig 2 shows the PCA for this subset. Comparison of the SOMAscan results between virally-infected patients and healthy controls from subset A by LIMMA revealed a total of 23 DEPs, 5 increased and 18 decreased (log_2_-fold change ≥ 1 and adjusted *p* < 0.01) (S3 Table).

**Fig 3:**
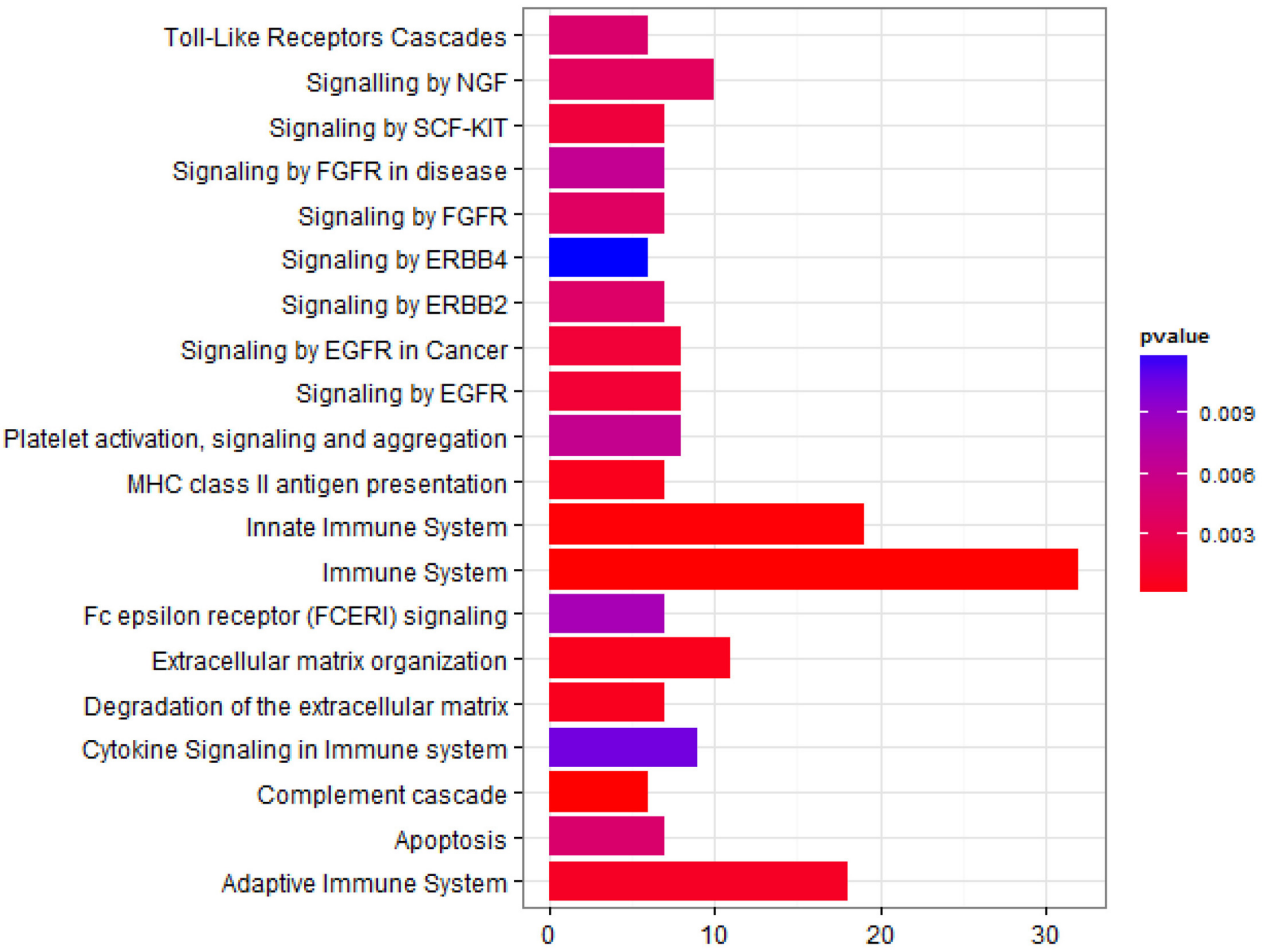
Functional analysis of DEPs from subset B. Pathway enrichment analysis for normalized log_2_–transformed expression values of proteins that were differentially expressed in IAV-positive versus IAV-negative patients from subset B is presented. Pathway terms that were enriched for the DEPs from subset B are indicated on the y-axis, the number of DEPs in the respective pathway category is indicated on the x-axis. The p-value for the probability that the observed distribution of expression occurred by chance is represented by colors of bars. The cut-off value for the pathway p-values was chosen at 0.05.

**Fig 4:**
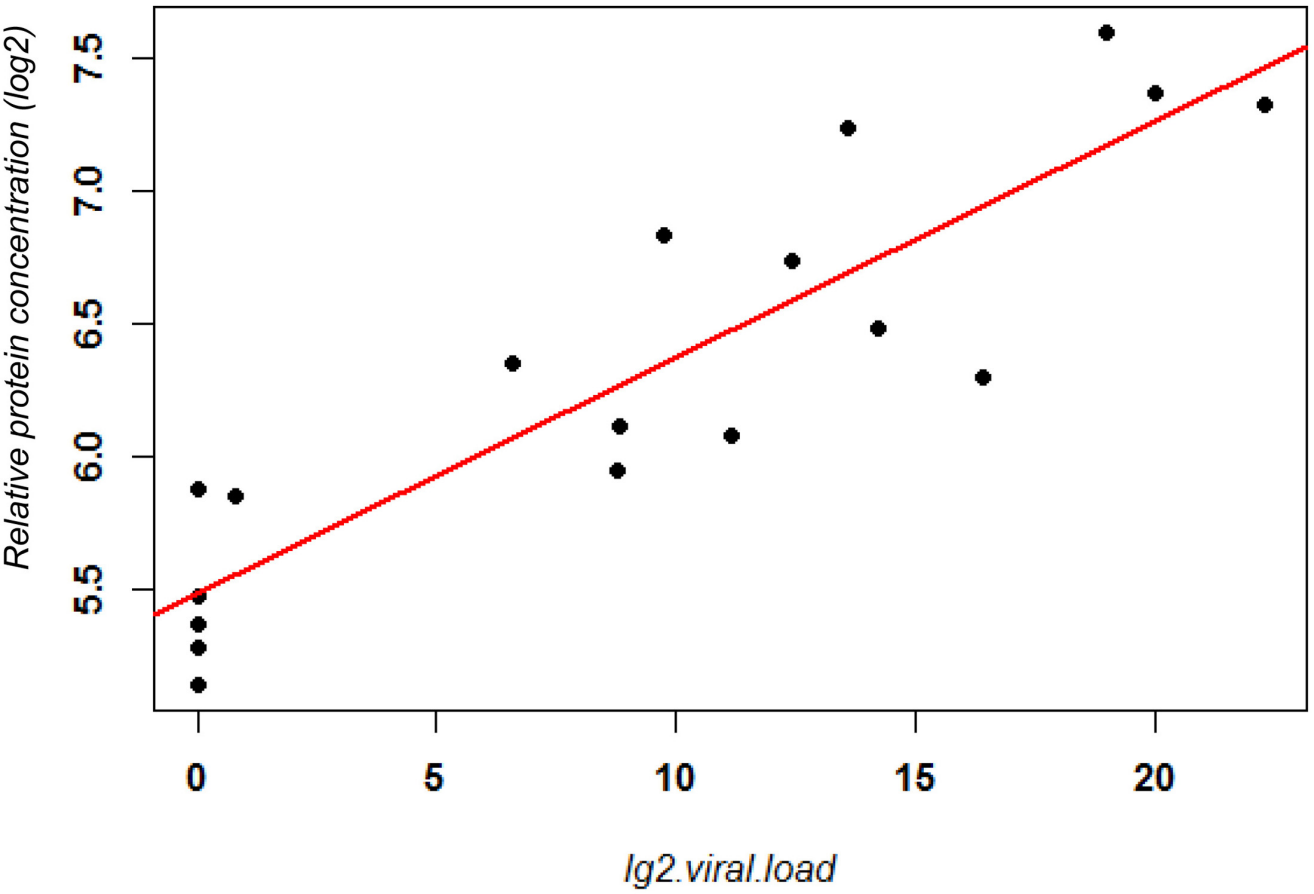
Scatter plot of RPS6KA5 expression levels and viral load. Scatter plot of normalized log_2_–transformed relative protein concentrations of RPS6KA5 which was the DEP that most strongly correlated with viral titers and log_2_-transformed virus titers are presented for patients from subset B. Red line: linear regression model.

In subset A, sample ID_4043 segregated separately in the PCA (Fig 2) from the other influenza virus-positive samples and had low levels of CXCL10 and IL6 (data not shown). CXCL10 and IL6 are known to be highly elevated after influenza infection [29], and the previous cytokine-chemokine analysis [28] indicated that plasma CXCL10 was correlated with viral loads. Therefore, we analyzed a second subset (subset B) which was identical to subset A except that the outlier sample ID_4043 was deleted. Thus, subset B (S2 Table) consisted of six healthy controls (same as in subset A) and 11 influenza-infected patients (12 minus 1 from subset A). The corresponding PCA showed good segregation between uninfected control and influenza virus-positive groups (S3 Fig). Accordingly, analyses of IL6 and CXCL10 expression levels showed significant differences between the two groups (Kruskal-Wallis, *p* < 0.01 and *p* < 0.05, respectively) (S4 Fig), even though samples from two healthy controls had relatively elevated CXCL10 compared to the other healthy controls. Further analysis of subset B using LIMMA revealed a total of 162 DEPs, 63 increased and 99 decreased in samples from virally infected patients compared to uninfected individuals (log_2_-fold change ≥ 1 and adjusted *p* < 0.01) (S4 Table). Functional analysis of these DEPs using the Reactome pathway database showed enrichment for pathways that are activated during the host immune response (Fig 3). Furthermore, 151 of the 162 (up- or down-regulated) DEPs were also highly correlated with viral load. 56 of the 151 DEPs were positively and 95 were negatively correlated with viral load (0.6 < ρ <—0.6 and FDR < 0.01) (Fig 4, S5 Table).

In conclusion, the SOMAscan analysis revealed the expected large heterogeneity among the 24 samples, similar to selective cytokine-chemokine multiplex assays, and reproduced results obtained previously with Luminex assays. In addition SOMAscan identified many additional DEPs that could not be detected in the previous study of the influenza cohort from which our samples were derived. Thus, SOMAscan is well suited for the detection of proteome differences in patients’ responses to influenza virus infection.

## Discussion

In this study, we demonstrate the utility of an unbiased, micro-proteomic analysis from small volumes of nasal washes to quantify proteome differences between influenza virus-infected patients and healthy individuals. SOMAscan identified more than 160 differentially expressed proteins (DEPs) from human respiratory influenza patients with a minimum viral load of 2^8^. Not surprising, most of the DEPs are associated with inflammation and/or immune response pathways. Among the 160 DEPs, 151 were correlated with viral loads in the respective subpopulation of influenza virus-infected patients

Among the DEPs with increased expression detected in our study, many are associated with the recruitment and activation of memory T cells. CXCL11 [31], CXCL10 [32, 33], IL-16 [34, 35], and GNLY (granulysisn) [36], are all chemokines chemotactic for activated and memory CD4 T cells, especially Th1 [33]. Another DEP that was correlated with viral load is amphiregulin. Amphiregulin is an epithelial and fibroblast cell mitogen and growth factor that, chronically, is also associated with inflammation [37, 38]. Recent results from lung influenza infection of mice point to a critical role for amphiregulin in limiting inflammation or otherwise protecting lung epithelium from damage after influenza virus infection [39–41]. Both innate lymphocytes (ILC) [41] and regulatory T cells [39] contributed to local amphiregulin in infected mouse lungs. It will be interesting to know what cells produce amphiregulin and whether it may be a biomarker to predict disease severity in human influenza patients.

The three DEPs with highest correlation to viral load in subset B were RPS6KA5 (MSK1, mitogen and stress activated kinase 1), KNG1 (kininogen), MAP2K1 (MEK-1, mitogen-activated protein kinase kinase-1), ANXA1 (annexin A1), and STIP1 (stress induced phosphoprotein 1). All of these proteins are associated with the initiation or resolution of inflammation and/or stress response and were strongly correlated with viral load. RPS6KA5 (MSK1) had the highest correlation with viral load in subset B (*rho* = 0.91, p < 0.01). MSK1 has been associated with activation [42–44] and inhibition of inflammation [44, 45] in lung epithelium. MSK1 is downstream of ERK1/2 and p38^MAPK^ and can phosphorylate NFκB p65 and is a key regulator of lung airway inflammation. MEK-1 is upstream of ERK1/2 and MSK-1 in the non-canonical pathway for p65/RelA phosphorylation by MSK1 leading to NFκB activated transcription of inflammatory cytokines and chemokines [46] including those detected as DEPs described above. MSK1 phosphorylation of p65/RelA is also critical for inflammatory cytokine production in an experimental model for respiratory syncytial virus (RSV) infection [47]. Herein is the first reported association of this key regulator of inflammation with respiratory influenza infection. Kininogen is a co-factor in the plasma kinin-kallikrein, contact system for blood coagulation and the generation of inflammatory mediators [48]. High molecular weight kininogen (HMWK) is precurosor for bradykinin, which is an important mediator of endothelial permeability. Hantavirus infected endothelial cells induce increased cleavage of HMWK with increased bradykinin and vascular permeability [49]. Increased nasal secretions have been correlated with increased glandular bradykinin in experimental infection of adult humans with IAV H1N1 [50]. Annexin A1 functions as a resolvin to reverse or resolve inflammation after it is initiated [51].

Most previous studies of respiratory influenza infection in humans have limited analyses of soluble proteins in serum or nasal washes to cytokines and chemokines predicted to be relevant to inflammation and immune response (reviewed in [52–54]). For example, the previous study of the large patient cohort from which our samples were derived [28] investigated changes in 41 cytokines and chemokines using Luminex. Distinct changes for IFNA2, CCL7 (MCP-3), IL6, and VEGFA (VEGF) in nasal washes and CCL7 (MCP-3), IL10, IL6, and CXCL10 (IP-10) in peripheral blood of human patients were significantly correlated with disease severity [28] but not viral load. IL6 was also identified as a DEP in our study; however, it should be noted that we used a more stringent *p =* 0.01. When *p =* 0.05, CCL7, IL6 and CXCL10 are also DEPs in our study. Another study compared plasma cytokines, metalloproteases, and complement activation in seven patients infected with H1N1 and found 16 proteins that were differentially expressed in the blood of influenza infected patients [29]. Five of the 16 proteins, CXCL10, IL6, IL17D, CSF2, PDGFB, from that study were also identified as DEPs in our study (adjusted *p* = 0.05). Moreover, elevated levels of CXCL10 (IP-10), IL6, IL17, and IL2 in sera of 16 hospital-admitted patients infected with H7N9 were previously reported, and IL6 and CXCL10 correlated with the 13 patients that developed severe disease [55]. A more recent study identified a correlation between serum levels of cytokines MIF, SCF, MCP-1, HGF, and SCGF-β and disease severity after H7N9 respiratory influenza infection [56]. We also found IL17D, IL6, and CXCL10 to be elevated DEPs in influenza virus-infected patients in our study.

A number of global proteomic analyses have been performed with influenza-infected cells *in vitro* [57–64] and mouse lungs ex vivo [65]. A recent global proteomic analysis of plasma proteins associated with influenza infection used two-dimensional gel electrophoresis and liquid chromatography mass spectrometry (LC-MS) [66]. Only FAM157A, leucine-rich alpha 2 glycoprotein, serum amyloid A protein, and dual oxidase 1 were statistically significantly different in plasma of healthy versus influenza-infected individuals by LC-MS.

In addition to up-regulated proteins, our study also identified many proteins with reduced expression in influenza virus-infected patients versus healthy controls. Of the proteins with reduced expression identified in our study, the five most-reduced DEPs in samples from influenza-infected patients that were also correlated with higher viral loads were CTSD, KLK7, MFGE8, MAPK9 and CD27, respectively. Cathepsin D (CTSD) is a lysosomal aspartic protease that has been shown to be highly expressed in alveolar resident macrophages and Kupffer cells following IAV infections in mice [67, 68]. KLK7 is a chymotrypsin-like serine protease that cleaves proteins at tyrosine, phenylalanine or leucine [69]. It is expressed in the epithelium of the upper and lower respiratory tract (nose, paranasal sinuses, larynx, trachea, bronchial tree) and in their submucosal glands but not the alveolar epithelium [70]. MFGE8 (lactadherin) is a cell adhesion protein that has been suggested to connect smooth muscle cells and elastic fibers of arteries [71]. Importantly, MFGE8 has been shown to promote opsonization of apoptotic cells and their subsequent phagocytosis through its phosphatidylserine (PS) and RGD integrin binding domains, respectively [72]. MAPK9 (JNK2) has been reported to play a role in p53 regulation and T cell differentiation and activation [73–75]. MAPKs and JNKs have been shown to play a role in the immune response to avian and human IAV infections [76–78]. CD27 is a member of the TNF-receptor family and serves as a T-cell costimulatory molecule [79]. In humans, CD27+ B cells exhibit a memory cell phenotype [80]. In T cells, CD27 is required for efficient CD4+ and CD8+ responses to influenza virus infection. This was only true for generating and maintaining antigen-specific T cell immunity, not effector cell expansion [81]. Of note, these reduced DEPs are involved in lung homeostasis and immunity and their reduced expression negatively correlates with viral load, suggesting that they could potentially be part of a biomarker or signature of infection severity. Importantly, none of these proteins are represented in other standard cytokine-chemokine multiplex panels typically used to analyze infected patient samples.

SOMAscan is capable of measuring a much larger number of cytokines and chemokines than are evaluable by standard techniques (Luminex and ELISA), especially with the limited sample volumes inherent in human translational studies. Additionally, other proteins, not known to contribute to inflammation, nor associated with inflammation or immunity are assessable. Therefore, SOMAscan allowed the identification of potentially novel pathways in the context of viral infections that may not be discovered with a pre-selected or biased multiplex assay such as Luminex and ELISA. While these proteins have reported functions, future studies could identify novel functions during viral infection that would provide additional insights into the complex host-influenza virus interactions.

The SOMAscan results were generally correlated well with Luminex and other assays that identified DEPs in mucosal fluids from influenza virus-infected patients. For example, IL-6, CXCL10/IP10, CCL7/MCP3, and CXCL8/IL-8 were among proteins present at high levels in nasal washes when measured by SOMAscan. Those proteins had similarly high relative levels in the same samples when measured by Luminex [28], but SOMAscan measured 25-fold more proteins. Among the proteins quantified by SOMAscan but not Luminex or other assay methods, many were highly correlated to influenza viral loads. As noted above, all of those proteins are integral to pathways important in inflammation and stress response. DEPs that segregated according to clinical disease severity were not identified. This result is not surprising since clinical disease severity was not correlated with viral loads in the patient samples assayed. Possibly serum protein levels measured by SOMAscan would have yielded correlation with disease severity since many of the clinical symptoms of disease are systemic. Also a larger patient sampling with more samples per disease severity group may yield significant correlations among proteins measured by SOMAscan and disease severity among influenza virus-infected patients. Finally, absolute correlation between different assay systems will always be compromised by differences in the method for collection of results, e.g., light absorbance, fluorescence intensity, etc., and differences in epitope recognition and binding for any given protein. The latter will be particularly relevant for antibody-dependent versus aptamer-dependent ligand binding. As noted in a recent commentary, SOMAscan will be no better or worse for detecting and quantifying a known protein.

The primary benefit provided by SOMAscan is in its discovery potential for identifying associations between proteins among a large catalog of proteins and a phenotype of interest [82]. It is within this context that we think the results from our study can move understanding of respiratory pathophysiology associated with influenza infection forward.

SOMAscan thus represents a very promising alternative methodology to existing technologies for the discovery of biomarkers for disease severity in respiratory infections including influenza. Furthermore, SOMAscan may also have great potential to analyze proteome changes within the mucosa in the context of other bacterial or viral respiratory infections or lung diseases in humans that are presently understudied because of limitations with other multiplex assay technologies.

## Materials and Methods

### Ethics statement

This study was conducted in compliance with Department of Health and Human Services regulations in 45 CFR46 and the Declaration of Helsinki. The Institutional Review Boards of St. Jude Children’s Research Hospital and the University of Tennessee Health Science Center/Le Bonheur Children’s Hospital approved the study. Nasal lavage samples were acquired from the St, Jude Children's Research Hospital Human Influenza Research Specimen Repository [28]. The samples were originally acquired after receiving written, informed consent from participants or the participants' parents/guardians. Use of the patient samples in the present study was approved by the UTHSC IRB (13-02691-XM).

### Patient samples

Subject inclusion criteria, definition of respiratory influenza infection, criteria for categorizing disease severity, and methods for sample collection and storage have been described [28]. Twenty-four samples from the repository were provided for the present study based solely upon the criteria of detection of influenza viral genome and disease severity after infection. Six samples per group were selected from each of four groups: i) uninfected healthy controls: individuals with no clinical signs or symptoms of respiratory infection and otherwise healthy; ii) mild: no emergency room (ER) visit or hospitalization; iii) moderate: ER visit but no hospitalization; and iv) severe: hospitalization. All of the assayed specimens were collected on the day of initial diagnosis (study day 0 in [28]). The uninfected, healthy controls were household contacts of infected patients who did not become infected with influenza. Patient demographics and viral loads determined by qRT-PCR are presented in S1 Table.

### SOMAscan analysis

The SOMAscan assay including calibration of the sample matrix for nasal lavage samples was performed by SomaLogic, Boulder, CO. Sixty μl of a 1:2 dilution of each nasal lavage supernatant was used for the SOMAscan assay.

SOMAmers are modified nucleic acid aptamers, each with both unique protein binding characteristics and unique identifying primary nucleic acid sequence that can be detected and quantified by DNA microarray. Each SOMAmer has been validated for its unique specificity, upper and lower limits of detection, and intra- and inter-assay variability. SOMAscan uses a 1,129 SOMAmer library to quantify 1,030 individual proteins or protein complexes that are detected by the library as relative fluorescence units of each SOMAmer bound to its respective protein [23]. Each SOMAmer has a unique specificity for a defined epitope. A given SOMAmer may thus bind to an isolated polypeptide or to a heterodimer of the same protein with another protein. Another SOMAmer may bind to the same heterodimer but neither polypeptide alone. Hence two different SOMAmers may be listed as detecting the same protein, but in fact bind to individual epitopes. Thus, the number of proteins (1,030) that can be detected is less than the total number of SOMAmers (1,129) used in the analysis. Details of SOMAmer validation, the list of proteins quantified by the current SOMAmer library, and SOMAscan assay procedure are at http://www.somalogic.com/Technology/SOMAscan-basic-info.aspx.

### Bioinformatics

Data were analyzed using the R software package [83]. Pre-processing steps included quantile normalization and log_2_ transformation of the signal intensity (relative light units) measures provided by the SOMAscan platform. Principal component analysis (PCA) analysis were performed using the affycoretools package [84]. Identification of differentially expressed probe sets (DEPs) was performed with the LIMMA package [85] using BH correction for multiple testing [86] using the indicated thresholds for fold-change and adjusted p-values (see results). Heatmaps were performed using the R software package [83]. GO, KEGG, Reactome enrichment analysis and was performed with the R package ReactomePA provided in the R package cluster Profiler [87].

## Supporting Information

S1 Fig Correlation of protein quantification by SOMAscan and Luminex. Correlation analysis of log_2_ relative fluorescence units (RFU) from SOMAscan and protein concentration (log_2_ of pg/ml) from Luminex study described by [28] are presented for IL6, CXCL10/IP-10, CCL7/MCP3, and CXCL8/IL8. Labels at top and left refer to individual chemokines and technology used for detection (SO: SOMAscan, LU: Luminex). The diagonal represents the histogram of the measured protein level determined by SOMAscan (SOMA) or Luminex (LUMI), the bottom left below the histogram diagonal represents scatter plots of pairs of measurements, the top right above the histogram diagonal presents correlation coefficients and p-values from a linear regression analysis of the respective pair-wise analyses. n = 24. **: *p* <0.01, ***: *p* < 0.001.
(PDF)

S2 Fig Distribution of viral loads. Upper panel: Histogram of log_2_-transformed viral loads showing the distribution of viral loads as measured by quantitative real-time reverse-transcription polymerase chain reaction at day 0 from all patients of the cohort [28] (n = 139 samples). The threshold of 256 (log_2_ = 8) is shown as a stippled line. Lower panel: Histogram of log_2_-transformed viral loads showing the distribution of viral loads as measured by quantitative real-time reverse-transcription polymerase chain reaction of the patients selected for the SOMAscan study (n = 24).
(PDF)

S3 Fig PCA analysis of normalized protein expression values for subset B. Principle component analysis (PCA) was performed with normalized log_2_–transformed protein expression values from nasal washes for 17 samples from the subset that were selected based on high vs. low viral loads and omitted outlier sample ID_4043 (subset B). The first two principal components are shown that represent 46% and 16%, respectively, of the total variation. Healthy control samples are labeled gray, and influenza virus-positive samples are labeled red. In addition, sample identities (e.g., ID_4002) are shown. Horizontal and vertical axis represent principle component 1 and 2, respectively.
(PDF)

S4 Fig Expression values for CXCL10 and IL6 from subset B. Normalized log_2_–transformed protein concentration of CXCL10 (A) and IL6 (B) for 17 samples from subset B that were selected based on high vs. low viral load. Bars represent mean expression values per group +/- 1 SEM. "negative": samples that were diagnosed IAV-negative; "positive": samples that were diagnosed IAV-positive. IL6 and CXCL10 expression levels showed significant differences between the two groups (Kruskal-Wallis, *p* < 0.01 and *p* < 0.05, respectively).
(PDF)

S1 Table Demographic data, type of influenza virus identified, log2 viral load, and relative severity of symptoms and clinical disease of respective patients. (DOCX)

S2 Table List of patients included into subsets A and B. (DOCX)

S3 Table List of proteins that were differentially expressed between uninfected control and influenza virus-positive patients from subset A. The patient identifiers are those from S1 Table. The numerical values for each patient column are the respective normalized log2 protein levels for each patient for each protein identified in column A and B. Log2 Fold Change (FC), Average (average respective protein level for all patients), t-statistic, *p*, adjusted *p*, and B were all calculated with LIMMA.
(TXT)

S4 Table List of proteins that were differentially expressed between uninfected control and influenza virus-positive patients from subset B. Column labels are the same as in S2 Table.
(TXT)

S5 Table List of DEPS from subset B that were correlated with viral load. cor is Spearman's correlation coefficient (ρ). (TXT)
