# Supplementary material for: Respiratory Mucosal Proteome Quantification in Human Influenza Infections
Source: PLoS One. 2016 Apr 18;11(4):e0153674. doi: 10.1371/journal.pone.0153674 (PMC4835085; doi:10.1371/journal.pone.0153674)
Supplement: S1 Fig — Correlation analysis of log2 relative fluorescence units (RFU) from SOMAscan and protein concentration (log2 of pg/ml) from Luminex study described by [28] are presented for IL6, CXCL10/IP-10, CCL7/MCP3, and CXCL8/IL8. Labels at top and left refer to individual chemokines and technology used for detection (SO: SOMAscan, LU: Luminex). The diagonal represents the histogram of the measured protein level determined by SOMAscan (SOMA) or Luminex (LUMI), the bottom left below the histogram diagonal represents scatter plots of pairs of measurements, the top right above the histogram diagonal presents correlation coefficients and p-values from a linear regression analysis of the respective pair-wise analyses. n = 24. **: p <0.01, ***: p < 0.001. (PDF) [file pone.0153674.s001.pdf]

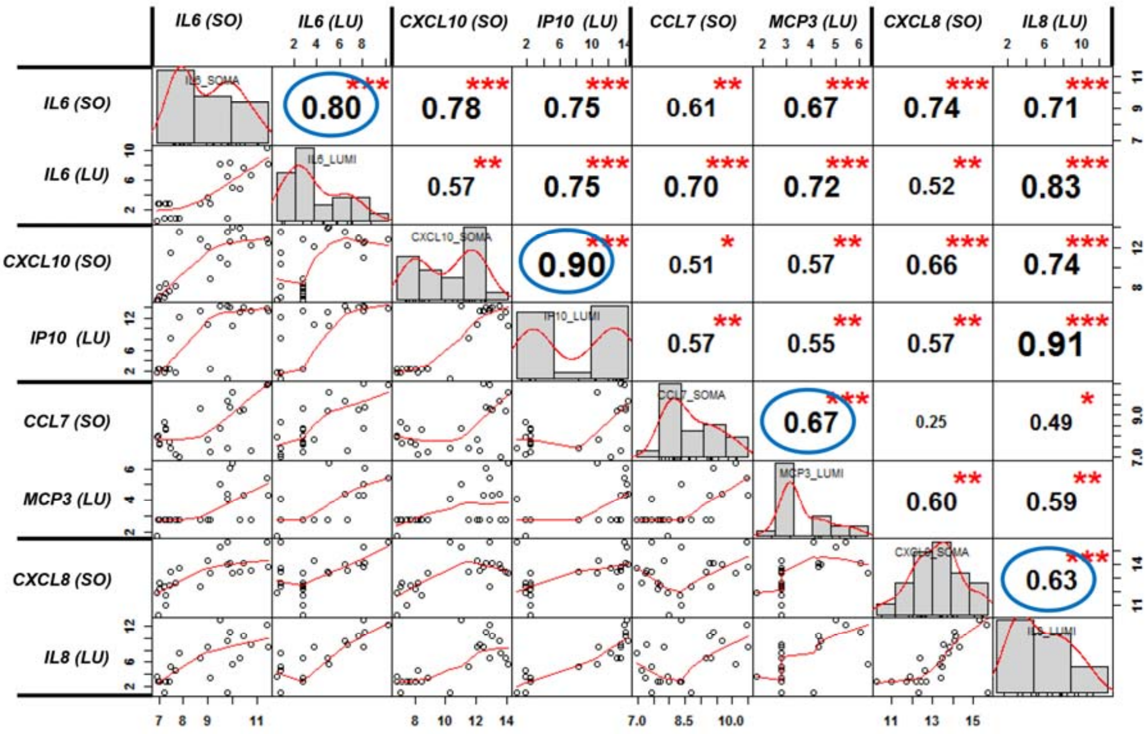

S1 Figure: Correlation of protein quantification by SOMAscan and Luminex

Correlation analysis of log_2 relative fluorescence units (RFU) from SOMAscan and protein concentration (log_2 of pg/ml) from Luminex study described by [1] are presented for IL6, CXCL10/IP-10, CCL7/MCP3, and CXCL8/IL8. Labels at top and left refer to individual chemokines and technology used for detection (SO: SOMAscan, LU: Luminex). The diagonal represents the histogram of the measured protein level determined by SOMAscan (SOMA) or Luminex (LUMI), the bottom left below the histogram diagonal represents scatter plots of pairs of measurements, the top right above the histogram diagonal presents correlation coefficients and p-values from a linear regression analysis of the respective pair-wise analyses. n=24. **: p <0.01, ***: p < 0.001.

1. Oshansky CM, Gartland AJ, Wong SS, Jeevan T, Wang D, et al. (2014) Mucosal Immune Responses Predict Clinical Outcomes during Influenza Infection Independently of Age and Viral Load. Am J Respir Crit Care Med 189: 449-462.
