# Supplementary material for: Respiratory Mucosal Proteome Quantification in Human Influenza Infections
Source: PLoS One. 2016 Apr 18;11(4):e0153674. doi: 10.1371/journal.pone.0153674 (PMC4835085; doi:10.1371/journal.pone.0153674)
Supplement: S2 Fig — Upper panel: Histogram of log2-transformed viral loads showing the distribution of viral loads as measured by quantitative real-time reverse-transcription polymerase chain reaction at day 0 from all patients of the cohort [28] (n = 139 samples). The threshold of 256 (log2 = 8) is shown as a stippled line. Lower panel: Histogram of log2-transformed viral loads showing the distribution of viral loads as measured by quantitative real-time reverse-transcription polymerase chain reaction of the patients selected for the SOMAscan study (n = 24). (PDF) [file pone.0153674.s002.pdf]

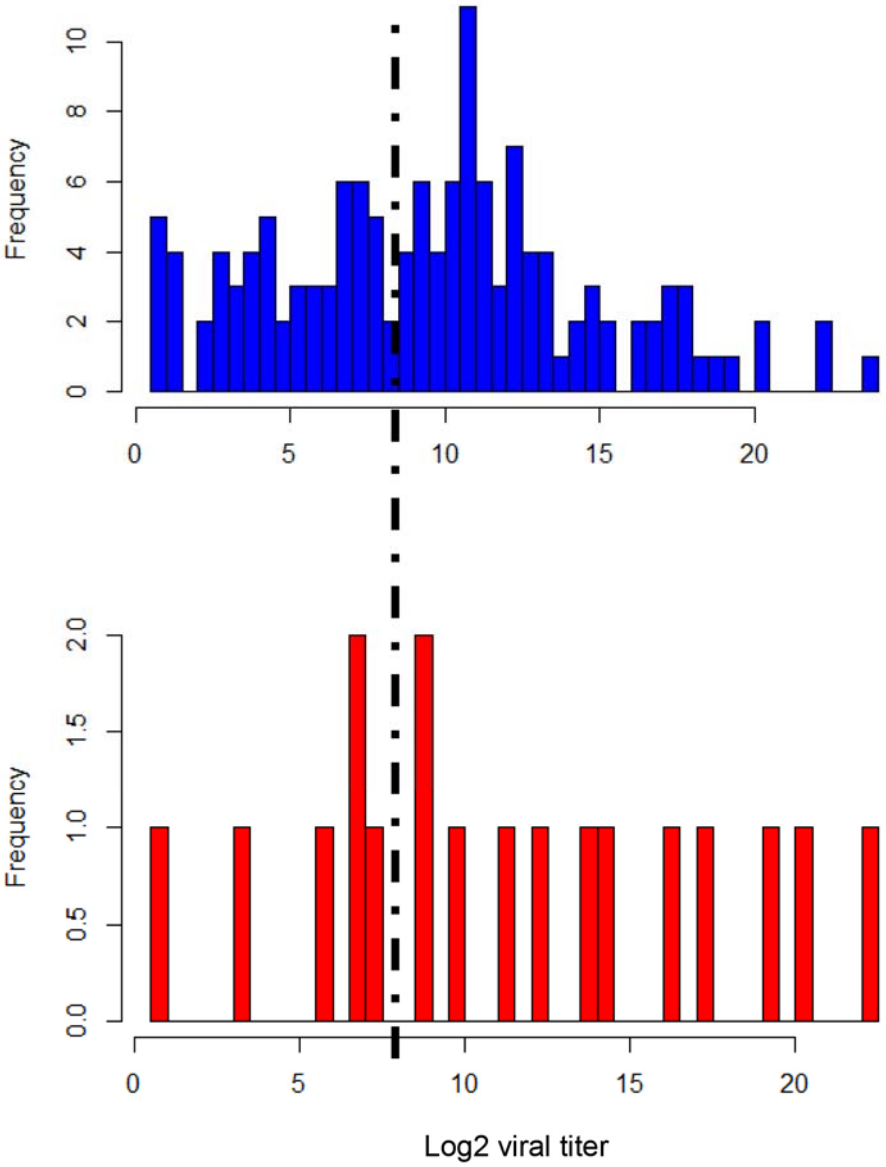

S2 Figure: Distribution of viral loads

Upper panel: Histogram of log_2-transformed viral loads showing the distribution of viral loads as measured by quantitative real-time reverse-transcription polymerase chain reaction at day 0 from all patients of the cohort [1] (n= 139 samples). The threshold of 256 ($log_2 = 8$) is shown as a stippled line. Lower panel: Histogram of log_2-transformed viral loads showing the distribution of viral loads as measured by quantitative real-time reverse-transcription polymerase chain reaction of the patients selected for the SOMAscan study (n = 24).

References

1. Oshansky CM, Gartland AJ, Wong SS, Jeevan T, Wang D, et al. (2014) Mucosal Immune Responses Predict Clinical Outcomes during Influenza Infection Independently of Age and Viral Load. Am J Respir Crit Care Med 189: 449-462.
