# Supplementary material for: Respiratory Mucosal Proteome Quantification in Human Influenza Infections
Source: PLoS One. 2016 Apr 18;11(4):e0153674. doi: 10.1371/journal.pone.0153674 (PMC4835085; doi:10.1371/journal.pone.0153674)
Supplement: S3 Fig — Principle component analysis (PCA) was performed with normalized log2–transformed protein expression values from nasal washes for 17 samples from the subset that were selected based on high vs. low viral loads and omitted outlier sample ID_4043 (subset B). The first two principal components are shown that represent 46% and 16%, respectively, of the total variation. Healthy control samples are labeled gray, and influenza virus-positive samples are labeled red. In addition, sample identities (e.g., ID_4002) are shown. Horizontal and vertical axis represent principle component 1 and 2, respectively. (PDF) [file pone.0153674.s003.pdf]

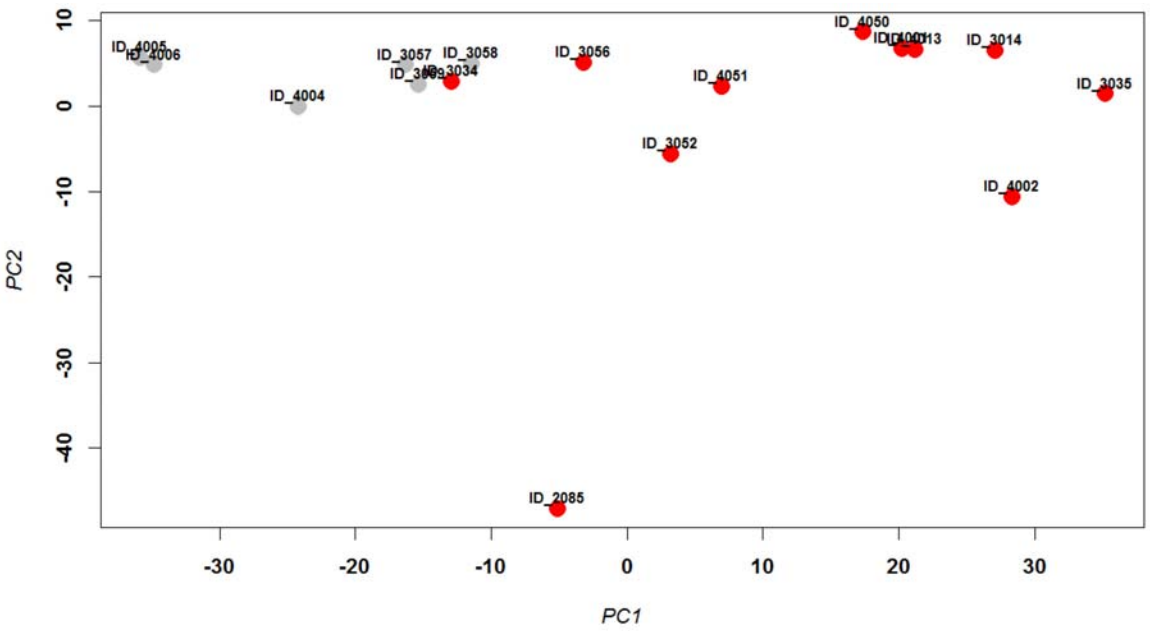

S3 Figure: PCA analysis of normalized protein expression values for subset B

Principle component analysis (PCA) was performed with normalized $\log_2$–transformed protein expression values from nasal washes for 17 samples from the subset that were selected based on high vs. low viral loads and omitted outlier sample ID_4043 (subset B). The first two principal components are shown that represent 46 % and 16 %, respectively, of the total variation. Healthy control samples are labeled gray, and influenza virus-positive samples are labeled red. In addition, sample identities (e.g., ID_4002) are shown. Horizontal and vertical axis represent principle component 1 and 2, respectively.
