# Supplementary material for: Respiratory Mucosal Proteome Quantification in Human Influenza Infections
Source: PLoS One. 2016 Apr 18;11(4):e0153674. doi: 10.1371/journal.pone.0153674 (PMC4835085; doi:10.1371/journal.pone.0153674)
Supplement: S4 Fig — Normalized log2–transformed protein concentration of CXCL10 (A) and IL6 (B) for 17 samples from subset B that were selected based on high vs. low viral load. Bars represent mean expression values per group +/- 1 SEM. "negative": samples that were diagnosed IAV-negative; "positive": samples that were diagnosed IAV-positive. IL6 and CXCL10 expression levels showed significant differences between the two groups (Kruskal-Wallis, p < 0.01 and p < 0.05, respectively). (PDF) [file pone.0153674.s004.pdf]

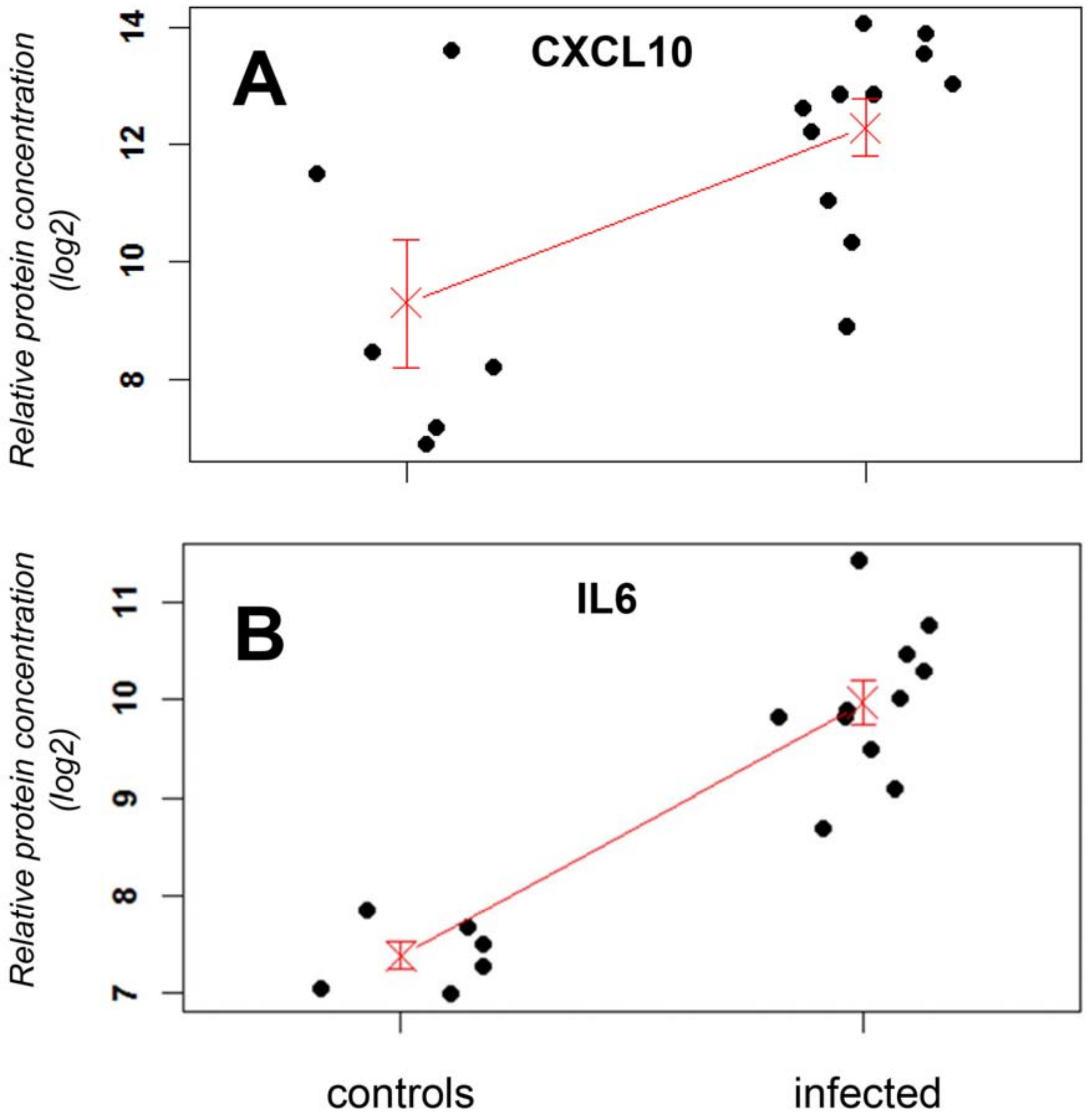

S4 Figure: Expression values for CXCL10 and IL6 from subset B

Normalized $\log_2$–transformed protein concentration of CXCL10 (A) and IL6 (B) for 17 samples from subset B that were selected based on high vs. low viral load. Bars represent mean expression values per group +/- 1 SEM. "negative": samples that were diagnosed IAV-negative; "positive": samples that were diagnosed IAV-positive. IL6 and CXCL10 expression levels showed significant differences between the two groups (Kruskal-Wallis, $p < 0.01$ and $p < 0.05$, respectively).
