# Supplementary material for: Respiratory Mucosal Proteome Quantification in Human Influenza Infections
Source: PLoS One. 2016 Apr 18;11(4):e0153674. doi: 10.1371/journal.pone.0153674 (PMC4835085; doi:10.1371/journal.pone.0153674)
Supplement: S1 Table — (DOCX) [file pone.0153674.s005.docx]

| **Table S1: Patient demographics and viral loads.** | | | | |  |
| --- | --- | --- | --- | --- | --- |
|  |  |  |  |  |  |
| **Sample ID** | **Gender** | **Age (yrs)** | **Influenza Virus Detected** | **Viral Load (log2)** | **Disease Severity** |
| ID_1026 | Male | 55 | A (pH1) | 7.1 | Severe |
| ID_2085 | Male | 0.3 | A (pH1) | 9.7 | Severe |
| ID_3001 | Male | 12 | A (H3) | -0.48 | Moderate |
| ID_3014 | Female | 0.4 | B | 14 | Moderate |
| ID_3019 | Female | 2.8 | A (H3) | 6.6 | Moderate |
| ID_3034 | Male | 9.7 | A (H3) | 8.8 | Severe |
| ID_3035 | Female | 1.9 | B | 19 | Severe |
| ID_3045 | Female | 3.7 | A (H3) | 2.8 | Mild |
| ID_3052 | Female | 28 | A (H3) | 14 | Mild |
| ID_3056 | Female | 8.3 | B | 11 | Mild |
| ID_3057 | Male | 13 | healthy control | 0 | N/A |
| ID_3058 | Female | 15 | healthy control | 0 | N/A |
| ID_3059 | Female | 33 | healthy control | 0 | N/A |
| ID_4001 | Female | 0.5 | A (H3) | 22 | Severe |
| ID_4002 | Male | 0.8 | A (H3) | 12 | Severe |
| ID_4004 | Female | 47 | healthy control | 0 | N/A |
| ID_4005 | Male | 52 | healthy control | 0 | N/A |
| ID_4006 | Female | 17 | healthy control | 0 | N/A |
| ID_4009 | Female | 25 | A (H3) | 6.5 | Mild |
| ID_4010 | Female | 12 | A (H3) | 5.6 | Mild |
| ID_4013 | Male | 2.1 | A (H3) | 16 | Mild |
| ID_4043 | Male | 3.4 | A (H3) | 17 | Moderate |
| ID_4050 | Female | 2.1 | B | 20 | Moderate |
| ID_4051 | Male | 3.8 | B | 8.7 | Moderate |
