# Supplementary material for: Respiratory Mucosal Proteome Quantification in Human Influenza Infections
Source: PLoS One. 2016 Apr 18;11(4):e0153674. doi: 10.1371/journal.pone.0153674 (PMC4835085; doi:10.1371/journal.pone.0153674)
Supplement: S2 Table — (DOCX) [file pone.0153674.s006.docx]

## Supplemental Table S2: List of patients included into subsets A and B.

**Subset A:**

Sample_ID Virus.detected lg2.viral.load

2 ID_2085 positive 9.745204

4 ID_3014 positive 13.577493

6 ID_3034 positive 8.828010

7 ID_3035 positive 19.016383

9 ID_3052 positive 14.217687

10 ID_3056 positive 11.153799

11 ID_3057 healthy 0

12 ID_3058 healthy 0

13 ID_3059 healthy 0

14 ID_4001 positive 22.292756

15 ID_4002 positive 12.449218

16 ID_4004 healthy 0

17 ID_4005 healthy 0

18 ID_4006 healthy 0

21 ID_4013 positive 16.412418

22 ID_4043 positive 17.168914

23 ID_4050 positive 20.029880

24 ID_4051 positive 8.756990

**Subset B:**

Sample_ID Virus.detected lg2.viral.load

2 ID_2085 positive 9.745204

4 ID_3014 positive 13.577493

6 ID_3034 positive 8.828010

7 ID_3035 positive 19.016383

9 ID_3052 positive 14.217687

10 ID_3056 positive 11.153799

11 ID_3057 healthy 0

12 ID_3058 healthy 0

13 ID_3059 healthy 0

14 ID_4001 positive 22.292756

15 ID_4002 positive 12.449218

16 ID_4004 healthy 0

17 ID_4005 healthy 0

18 ID_4006 healthy 0

21 ID_4013 positive 16.412418

23 ID_4050 positive 20.029880

24 ID_4051 positive 8.756990
